# The Effect of the Nurse-Led Mental Health Empowerment Programme on Adolescents’ Mental Health Literacy, Well-Being and Stress Coping Levels: Study Protocol

**DOI:** 10.1192/j.eurpsy.2026.11821

**Published:** 2026-07-31

**Authors:** G. Öztürk, A. Ergün

**Affiliations:** 1 Nursing, Fenerbahce University; 2 Nursing, Marmara University, Istanbul, Türkiye

## Abstract

**Introduction:**

Adolescence is a sensitive developmental period with increased risk for stress and mental health problems. Schools are important community settings for prevention and health promotion. Nurse-led, school-based interventions may provide an effective and sustainable approach.

**Objectives:**

The aim of this study is to evaluate the effectiveness of a Mental Health Empowerment Programme on adolescents’ mental health literacy, competencies, coping strategies, and well-being.

**Methods:**

A quasi-experimental pre–post-follow- up controlled design will be carried out with 102 adolescents aged 15–16 years from a public high school in Istanbul, Türkiye. Participants will be assigned by class clusters to intervention and control groups. The intervention includes six weekly modules adapted from an evidence-based mental health empowerment programme and a booster session at three months. Data will be collected at baseline, post-intervention, and at three-month follow-up.

**Results:**

Recruitment and baseline data collection for pilot study will start in January 2026. It is expected that the intervention group will show greater improvements in literacy, competencies, coping, and well-being compared to the control group.

**Conclusions:**

This study protocol describes one of the first nurse-led school-based mental health empowerment programmes for adolescents in Türkiye. The results are expected to support the integration of literacy, first aid, and stress management into school health practice and to highlight the role of nurses in promoting adolescent mental health.

**Disclosure of Interest:**

None Declared

